# Potential Drug-Drug Interactions With Nirmatrelvir/Ritonavir Among Long-Term Care Facility Residents

**DOI:** 10.1016/j.jamda.2026.106365

**Published:** 2026-07-07

**Authors:** Marzan A. Khan, Lori A. Daiello, Sarah D. Berry, Daniel A. Harris, Hemalkumar B. Mehta, Yu-Chia (Sam) Hsu, Charles E. Leonard, Kevin W. McConeghy, Andrew R. Zullo

**Affiliations:** a Department of Epidemiology, Brown University School of Public Health, Providence, RI, USA; b Center for Gerontology and Healthcare Research, Brown University School of Public Health, Providence, RI, USA; c Department of Neurology, Alpert Medical School of Brown University, Providence, RI, USA; d Hinda and Arthur Marcus Institute for Aging Research and Department of Medicine, Hebrew Senior Life, Boston, MA, USA; e Department of Epidemiology, College of Health Sciences, University of Delaware, Newark, DE, USA; f Center for Drug Safety and Effectiveness, Johns Hopkins Bloomberg School of Public Health, Baltimore, MD, USA; g Department of Epidemiology, Johns Hopkins Bloomberg School of Public Health, Baltimore, MD, USA; h Center for Real-World Effectiveness and Safety of Therapeutics, University of Pennsylvania Perelman School of Medicine, Philadelphia, PA, USA; i Department of Biostatistics, Epidemiology, and Informatics, Perelman School of Medicine, University of Pennsylvania, Philadelphia, PA, USA; j Transformative Health Systems Research to Improve Veteran Equity and Independence Center of Innovation (THRIVE COIN), Veterans Affairs Providence Health Care System, Providence, RI, USA; k Department of Health Services, Policy and Practice, Brown University School of Public Health, Providence, RI, USA; l Department of Pharmacy, Brown University Health, Rhode Island Hospital, Providence, RI, USA

**Keywords:** COVID-19, drug-drug interaction, long-term care facility residents, nirmatrelvir/ritonavir

## Abstract

**Objectives:**

Nirmatrelvir/ritonavir (Paxlovid) is an oral antiviral for COVID-19. Although effective, its use is complicated by clinically important potential drug-drug interactions (DDIs). Long-term care facility (LTCF) residents are especially vulnerable to both COVID-19 and the adverse effects of potential DDIs because of advanced age, multimorbidity, and polypharmacy. The objective of this study was to quantify the prevalence and duration of potential DDIs among US LTCF residents administered nirmatrelvir/ritonavir.

**Design:**

We conducted a cohort study of LTCF residents using electronic medication administration record data from the Long-Term Care Data Cooperative linked to Medicare claims (January 2022-January 2025).

**Methods:**

Days of nirmatrelvir/ritonavir administration were identified, and concurrent exposure to 236 potentially interacting medications was assessed. A potential DDI was defined as same-day coadministration, reflecting concurrent exposure rather than clinical adverse effects. We estimated the prevalence of potential DDIs and the median duration of overlapping exposure.

**Results:**

Among 48,260 LTCF residents treated with nirmatrelvir/ritonavir, the mean age was 78.8 years (SD, 10.9), 58% were female, and 76% were non-Hispanic White. Overall, 86% were exposed to at least 1 potential DDI. The most common potentially interacting medications were atorvastatin (28.1%; 95% confidence limits [CLs], 27.7-28.50), amlodipine (21.6%; 95% CLs, 21.2-21.9), and apixaban (15.9%; 95% CLs, 15.6-16.3). Median days of overlapping exposure ranged from 5 to 6 days.

**Discussion:**

Potential DDIs most frequently involve medications requiring temporary withholding or dose adjustment rather than contraindication. The high prevalence of potential DDIs likely reflects the complexity of medication management in LTCFs, where antiviral use must be weighed against comorbidities and polypharmacy.

**Conclusions and Implications:**

Potential DDIs with nirmatrelvir/ritonavir were common among LTCF residents, though contraindicated ones were uncommon. These findings underscore the need for ongoing medication reviews and clinician and pharmacist oversight to ensure safe antiviral use in LTCFs

Long-term care facility (LTCF) residents are at increased risk for COVID-19 because of a convergence of multiple individual and setting-specific factors. Advanced age, multiple chronic medical conditions, congregate living, and reliance on staff caregivers are aspects that facilitate virus transmission and increase the incidence of severe disease outcomes.^1^ Nirmatrelvir/ritonavir (Paxlovid) is an oral antiviral for the treatment of mild-to-moderate COVID-19.^2^ When initiated early in the course of illness, nirmatrelvir/ritonavir may have substantial benefits for LTC residents as it is associated with reductions in duration of COVID-19 infection,^3^ symptom severity, decreased hospitalization,^4^ and mortality rates.^5^ Despite its effectiveness, nirmatrelvir/ritonavir may be underutilized in LTC settings because of important clinical considerations that contraindicate (eg, severe liver or kidney disease) or complicate (eg, numerous drug interactions) prescribing.

Nirmatrelvir halts severe acute respiratory syndrome coronavirus 2 viral replication by blocking a key enzyme required for viral protein processing, whereas ritonavir increases blood plasma concentrations of nirmatrelvir through inhibition of the cytochrome P450 3A4 (CYP3A4) enzyme. LTC residents are frequently prescribed complex medication regimens that include 1 or more drugs metabolized by CYP3A4, such as statins, calcium channel blockers, and psychotropic agents. Coadministration with nirmatrelvir/ritonavir may lead to elevated plasma concentrations of other medications that also utilize this pathway, increasing the risk of clinically relevant potential drugdrug interactions (DDIs) and related adverse events.^6^ In LTCFs, where medication changes often require coordination across prescribers, nursing staff, and consultant pharmacists, potential DDIs may pose challenges for timely antiviral use and safe medication management.

Although LTC residents are at greater risk for hospitalization and death from COVID-19 than community-dwelling older adults,^7^ clinicians may be reluctant to prescribe nirmatrelvir/ritonavir because of safety concerns about potential DDI-mediated adverse outcomes^8^ in a population characterized by advanced age, multimorbidity, and polypharmacy. Therefore, the objective of this study was to investigate the prevalence and duration of concurrent use of medications that may potentially interact with nirmatrelvir/ritonavir among LTC residents in the United States to inform safer clinical decisionmaking.

## Methods

### Study Design and Data Sources

We conducted a cohort study comprising LTCF residents identified from the Long-Term Care Data Cooperative,^9^ a novel data repository of electronic health records from skilled nursing facilities and nursing homes across the United States. We used electronic medication administration records (eMARs) between January 1, 2022 and January 31, 2025 to derive the cohort. Demographics, including sex, race, and date of birth, were obtained from the 2009 to 2024 Medicare Master Beneficiary Summary Files, after linkage to the eMARs. In addition, we used the Long-Term Care Data Cooperative conditions, stays, and episodes files for information on comorbid conditions and LTCF residence.

The Brown University Institutional Review Board approved this study. Analyses were performed using SAS, version 9.4 (SAS Institute, Inc). Code and documentation are available on GitHub: https://doi.org/10.5281/zenodo.17245374.

### Study Population

We included nirmatrelvir/ritonavir administrations from the eMAR file, excluding those that occurred before 2022, with missing dates, or with implausible routes (eg, gastric, rectal, or unknown). If multiple administrations occurred on the same day for any resident, only 1 record was retained, as the primary unit of analysis was the residentday of nirmatrelvir/ritonavir administration (Supplementary Figure 1). A small subset (6.5%) of residents lacked Medicare enrollment data. We retained them in the analytic sample to maximize the number of individuals for whom exposure to potential DDIs could be observed, and because we did not adjust for covariates, exclusion because of missing demographic information was unnecessary.

### Measures

#### Definition of potential DDIs

We used the National Institutes of Health (NIH) list of 236 medications (February 2024 version) categorized by potential interaction severity (Supplementary Table 1).^10^ These medications were identified by an expert panel as potentially interacting with nirmatrelvir/ritonavir. Because this study assessed concurrent administration rather than clinical consequences, we refer throughout to potential DDIs. We considered any day on which an interacting medication and nirmatrelvir/ritonavir were coadministered, at any dose or frequency, as a day when a potential DDI occurred. We used same-day coadministration as an operational definition because eMAR data permit reliable identification of medications administered concurrently on the same day.

Nirmatrelvir/ritonavir administrations separated by ≥14 days were considered distinct courses. Medication dose, strength, generic, and brand name variables were used to categorize dosage.

### Resident characteristics

Comorbid conditions were defined as the presence of at least 1 diagnosis code. We selected a limited set of common, clinically relevant chronic conditions contributing to medication use and clinical complexity in long-term care populations. LTCF length of stay was defined as days of residence prior to first nirmatrelvir/ritonavir administration. Polypharmacy was defined as the number of distinct medications administered during the 180 days before the first nirmatrelvir/ritonavir administration.

### Statistical analysis

We reported descriptive statistics on age at the time of first nirmatrelvir/ritonavir administration during the study period, along with time-invariant characteristics (sex and race), both overall and stratified by exposure to at least 1 potential DDI. We also summarized selected clinical characteristics, including comorbid conditions, polypharmacy, and LTCF length of stay. We assessed the prevalence of potential DDIs as the percentage of LTCF residents ever exposed to an interacting medication among the total number of LTCF residents exposed to nirmatrelvir/ritonavir with 95% Clopper-Pearson exact confidence limits (CLs). The duration of exposure was computed as the median number of days LTCF residents were exposed to a particular potential DDI.

## Results

### Study Population

Among a total of 1,440,098 LTCF residents who received any medication, 48,260 received 481,893 administrations of nirmatrelvir/ritonavir between January 1, 2022 and January 31, 2025. After limiting to 1 administration record per resident per day, there were 263,461 resident-days of nirmatrelvir/ritonavir administrations. The standard dose of nirmatrelvir/ritonavir (300 mg nirmatrelvir/100 mg ritonavir) was administered to 70% of residents, whereas the remainder received a reduced dose. Among 35,514 standard-dose courses, 7% were administered to residents with chronic kidney disease. The mean age of the residents who received nirmatrelvir/ritonavir administrations was 78.8 (SD, 10.9) years, 58% of whom were female, and 76% were non-Hispanic White. LTCF residents with at least 1 potential DDI (85.9%) were younger (median age, 79 vs 82 years old) and less likely to be female (56.6% vs 63.2%) compared with those with no potential DDI. The prevalence of selected chronic conditions was 31.4% for diabetes, 20.9% for diagnosed dementia, 22.9% for heart failure, and 30% for chronic kidney disease (Supplementary Table 2). About 5% of the residents received more than 1 course of nirmatrelvir/ritonavir treatment―the median (Q1, Q3) number of courses was 2 (2, 2), and the maximum was 4. Nirmatrelvir/ritonavir administrations peaked in December 2022 and December 2023, when 7.2% and 6.6% of the total 481,893 nirmatrelvir/ritonavir administrations, respectively, were observed (Supplementary Figure 2).

Residents were administered a mean (SD) of 1.8 (5.8) medications in the 180 days before nirmatrelvir/ritonavir initiation in the LTCF and a mean (SD) of 2.1 (1.5) potentially interacting medications during nirmatrelvir/ritonavir treatment. The median (Q1, Q3) LTCF stay prior to nirmatrelvir/ritonavir initiation was 13 (0, 47) days. In addition, 16.2% of residents were classified as long stay. Among all residents treated with nirmatrelvir/ritonavir, 60.9% had at least 1 treatment day with ≥2 potentially interacting medications, and 35.3% had at least 1 day with ≥3. In addition, several CYP3A4 inhibitors other than nirmatrelvir/ritonavir were also observed during treatment, including diltiazem (3.8%), amiodarone (2.4%), verapamil (0.5%), erythromycin (0.3%), and ketoconazole (1.6%) (Supplementary Table 3).

### Potential DDI Quantification

Atorvastatin, which NIH guidelines recommend withholding if clinically appropriate, was the most frequently coadministered medication, administered to 13,543 residents (28.1% [95% CLs, 27.7%-28.5%]) with a median (Q1, Q3) exposure duration of 5 (2, 6) days. Amlodipine (21.6% [95% CLs, 21.2%-21.9%]; median, 5 [5, 6] days) and apixaban (15.9% [95% CLs, 15.6%-16.3%]; median, 6 [4, 6] days), both requiring dose adjustment and monitoring, were also highly prevalent. Other clinically important potentially interacting medications included psychotropics commonly used in LTCFs―such as trazodone (10.8%), mirtazapine (10.1%), quetiapine (5.6%), and risperidone (3.2%)―which generally require dose adjustment or monitoring. Additional medications included clopidogrel (8.8%), for which an alternative is recommended, and medications more commonly managed through temporary withholding, including salmeterol (3.4%), rosuvastatin (3.1%), and simvastatin (2.8%). Most of these medications had a median exposure duration of 5 days (Table 1). Atorvastatin, amlodipine, and apixaban, when combined, accounted for nearly two-thirds of all potentially interacting medication administrations.

Statistical measures of all 142 potentially interacting medications we observed in the data are shown in Supplementary Table 4.

## Discussion

In this large, nationally representative cohort of US LTCF residents treated with nirmatrelvir/ritonavir, 86% were administered at least 1 potentially interacting medication. Most potential DDIs involved medications requiring temporary withholding or dose adjustment rather than absolute contraindication, such as atorvastatin, amlodipine, apixaban, and tamsulosin. These patterns align with a study of veterans from community living centers, which similarly identified atorvastatin, amlodipine, and tamsulosin as commonly prescribed with nirmatrelvir/ritonavir.^11^ Clopidogrel, salmeterol, and several psychotropic agents (including trazodone, mirtazapine, quetiapine, and risperidone) as well as lipid-lowering agents (rosuvastatin and simvastatin) were less frequent. The prevalence of atorvastatin is notable as ritonavir’s potent CYP3A4 inhibition can markedly increase atorvastatin levels, up to 5-fold after 5 days of coadministration.^12^ A consequence can be statin-associated myalgia or rhabdomyolysis (the most severe form of myalgia), particularly when interacting medications are not withheld during treatment.^13^ Exposure to multiple potentially interacting medications was common, with 60.9% of residents having ≥2 potentially interacting medications on at least 1 treatment day, underscoring the complexity of medication management during antiviral prescribing.

The high prevalence of potential DDIs likely reflects operational challenges in LTCFs. Unlike hospitals, most LTCFs have a shortage of on-site prescribers or pharmacists, and medication orders are often entered by nurses for off-site clinicians to cosign. Monitoring by pharmacists, especially at night, is scarce, and adjusting chronic medications for a brief, 5-day course can be difficult in facilities experiencing staffing shortages. In these settings, a provider may relay a nirmatrelvir/ritonavir order, nursing staff transcribe and administer the drug, and later reconcile interacting medications once a potential DDI alert appears. Such workflow constraints help explain why potential DDIs are so prevalent. Although McGarry et al^14^ found low rates of nirmatrelvir/ritonavir prescribing in LTCFs, these low rates may partly reflect concern about the challenges of managing extensive potential DDIs in LTCFs. In addition, clinicians may give priority to the short-term benefits of antiviral therapy when they believe these outweigh the risk of potential DDIs. However, ritonavir irreversibly inhibits CYP3A4, which can extend the period of potential interaction beyond 5 days.^6^

A major strength of this study is the use of eMAR, which captures drugs administered rather than prescribed, allowing precise measurement of overlapping exposure time. We report statistical measures capturing both early and recent periods of nirmatrelvir/ritonavir use in the United States. LTCFs provide a comprehensive assessment of real-world administration patterns and potential DDIs to inform clinical practice. However, we did not assess the clinical outcomes associated with potential DDIs, and we could not determine how often clinicians and nursing staff followed recommended treatment guidelines. Evaluating whether potential DDIs resulted in realized adverse clinical outcomes (eg, bleeding, arrhythmias, falls, myopathy, or hospitalization) warrants a separate outcomes-focused investigation. In addition, because potential DDIs were defined using same-day coadministration, this approach may underestimate interaction risk extending beyond same-day overlap. Our findings support ongoing routine medication review at the time of nirmatrelvir/ritonavir prescribing, continued pharmacist involvement in LTCF antiviral management, and future studies evaluating whether potential DDIs translate into adverse clinical outcomes.

## Conclusions and Implications

Potential DDIs with nirmatrelvir/ritonavir were highly prevalent among LTCF residents, with many exposed to multiple interacting medications during treatment. These interactions commonly involved statins, cardiovascular therapies, anticoagulants, and psychotropic medications. Although contraindicated potential interactions were uncommon, most involved medications requiring temporary withholding, dose adjustment, or monitoring, highlighting the need for active medication management during antiviral prescribing. In LTCFs, where medication changes require coordination across prescribers, nursing staff, and pharmacists, these findings underscore the importance of structured medication review processes and adequate staffing to support safe antiviral use. Future research should evaluate whether these potential interactions result in adverse clinical outcomes and identify strategies to optimize medication management in this setting.

## Supplementary Material

Supplementary Material

Supplementary data related to this article can be found online at https://doi.org/10.1016/j.jamda.2026.106365.

## Figures and Tables

**Table 1:** Top 20 Prevalent Interacting Medications With Nirmatrelvir/Ritonavir and Exposure Duration Among LTCF Residents, 2022 to 2025

Rank	Medication	NIH recommendation*	Number of Residents	Prevalence as a Percentage (95% CLs)†	Median Days of Potential DDI Exposure (Q1, Q3)
1	Atorvastatin	Temporarily withhold concomitant medication, if clinically appropriate	13,543	28.1 (27.7-28.5)	5 (2, 6)
2	Amlodipine	Adjust concomitant medication dose and monitor for adverse effects	10,404	21.6 (21.2-21.9)	5 (5, 6)
3	Apixaban	Adjust concomitant medication dose and monitor for adverse effects	7687	15.9 (15.6-16.3)	5 (4, 6)
4	Tamsulosin	Adjust concomitant medication dose and monitor for adverse effects	5699	11.8 (11.5-12.1)	5 (2, 6)
5	Trazodone	Adjust concomitant medication dose and monitor for adverse effects	5216	10.8 (10.5-11.1)	5 (5, 6)
6	Mirtazapine	Continue concomitant medication and monitor for adverse effects	4882	10.1 (9.8-10.4)	5 (5, 6)
7	Hydrocodone	Adjust concomitant medication dose and monitor for adverse effects	4563	9.5 (9.2-9.7)	4 (2, 5)
8	Clopidogrel	Prescribe alternative COVID-19 therapy	4238	8.8 (8.5-9.0)	5 (5, 6)
9	Oxycodone	Adjust concomitant medication dose and monitor for adverse effects	4131	8.6 (8.3-8.8)	4 (2, 5)
10	Tramadol	Continue concomitant medication and monitor for adverse effects	3575	7.4 (7.2-7.6)	5 (2, 6)
11	Dexamethasone	Continue concomitant medication and monitor for adverse effects	3246	6.7 (6.5-7.0)	5 (3, 5)
12	Buspirone	Adjust concomitant medication dose and monitor for adverse effects	2972	6.2 (5.9-6.4)	5 (5, 6)
13	Quetiapine	Adjust concomitant medication dose and monitor for adverse effects	2703	5.6 (5.4-5.8)	5 (4, 6)
14	Diltiazem	Adjust concomitant medication dose and monitor for adverse effects	1855	3.8 (3.7-4.0)	5 (5, 6)
15	Oxybutynin	Continue concomitant medication and monitor for adverse effects	1689	3.5 (3.3-3.7)	5 (5, 6)
16	Salmeterol	Temporarily withhold concomitant medication, if clinically appropriate	1624	3.4 (3.2-3.5)	5 (4, 6)
17	Risperidone	Continue concomitant medication and monitor for adverse effects	1531	3.2 (3.0-3.3)	5 (5, 6)
18	Rosuvastatin	Temporarily withhold concomitant medication, if clinically appropriate	1479	3.1 (2.9-3.2)	5 (2, 6)
19	Simvastatin	Temporarily withhold concomitant medication, if clinically appropriate	1362	2.8 (2.7-3.0)	4 (1, 5)
20	Alprazolam	Adjust concomitant medication dose and monitor for adverse effects	1290	2.7 (2.5-2.8)	5 (3, 6)

Q1, lower quartile; Q3, upper quartile.

Of a total of 50,832 distinct courses of nirmatrelvir/ritonavir (defined by a gap period of ≥14 days between administrations per resident) during the study period, 1373 (2.7%) showed a spillover pattern with 1 dose administered on the day before, and the following dose administered on the final day. In addition, about 5% of the 48,260 unique residents received more than 1 course of nirmatrelvir/ritonavir treatment. Together, these patterns provide context for why the upper quartile of potential DDI exposure days was 6 for most of the medications.

* The NIH recommendation column reflects the recommended clinical management category of each potentially interacting medication, including recommendations to temporarily withhold, dose adjust and monitor, continue and monitor, or use alternative COVID-19 therapy.

† The prevalence of interacting medications is expressed as a percentage.
