# Supplementary material for: Potential Drug-Drug Interactions With Nirmatrelvir/Ritonavir Among Long-Term Care Facility Residents
Source: J Am Med Dir Assoc. Author manuscript; Available in PMC 2026 Aug 6. (PMC13439577; doi:10.1016/j.jamda.2026.106365)
Supplement: Supplementary file 1 [file NIHMS2194683-supplement-Supplementary_Material.docx]

**Supplemental Content**

**Potential drug-drug interactions with nirmatrelvir/ritonavir among long-term care facility residents.**

**Supplementary Figure S1: Flowchart showing eligible nirmatrelvir/ritonavir administrations among long-term care facility residents, 2022-2025.**

All nirmatrelvir/ritonavir administrations in the eMAR file

N=482,584

Unique residents, n=48,316

Administrations between January 1, 2022 and January 31, 2025

N= 481,893

Unique residents, n=48,260

1. Administration date and time missing or occurred before 2022

N=134

2. Administration route listed as gastric, rectal, or unknown

N=557

One administration per resident per day between January 1, 2022 and January 31, 2025

N= 263,461

Unique residents, n=48,260

Other same-day administrations, per resident

N=218,432

All medication administrations in the eMAR file

N=69,032,954

Unique residents, n=1,440,098

Other medications besides nirmatrelvir/ritonavir administrations

N=68,550,370

Excluded

Excluded

Excluded

N=Total number of administrations

n=Number of unique residents

Abbreviation: eMAR=Electronic Medication Administration Record, LTCDC=Long-Term Care Data Cooperative

Note: Of the 48,260 residents, 3,129 (6.5%) had missing demographic information because they could not be linked to Medicare data or had no available records in the annual Master Beneficiary Summary Files (2009-2024).

**Supplementary Table S1: Drugs with potential interactions with nirmatrelvir/ritonavir, including generic and brand names.**

| **Generic name** | **Brand name 1** | **Brand name 2** | **Brand name 3** | **Brand name 4** | **Brand name 5** | **Brand name 6** | **Brand name 7** | **Brand name 8** | **Brand name 9** |
| --- | --- | --- | --- | --- | --- | --- | --- | --- | --- |
| **Prescribe Alternative COVID-19 Therapy** | | | | | | | | | |
| **Anticonvulsants** |  |  |  |  |  |  |  |  |  |
| Carbamazepine | Tegretol | Carbatrol | Epitol | Equetro |  |  |  |  |  |
| Phenobarbital | Luminal | Solfoton | Sezaby |  |  |  |  |  |  |
| Phenytoin | Dilantin | Phenytek |  |  |  |  |  |  |  |
| Primidone | Mysoline |  |  |  |  |  |  |  |  |
| **Anti-Infectives** |  |  |  |  |  |  |  |  |  |
| Glecaprevir/pibrentasvir | Mavyret |  |  |  |  |  |  |  |  |
| Rifampin | Rifadin | Rimactane |  |  |  |  |  |  |  |
| Rifapentine | Priftin |  |  |  |  |  |  |  |  |
| **Immunosuppressants** |  |  |  |  |  |  |  |  |  |
| Voclosporin | Lupkynis |  |  |  |  |  |  |  |  |
| **Cardiovascular** |  |  |  |  |  |  |  |  |  |
| Amiodarone | Cordarone | Pacerone |  |  |  |  |  |  |  |
| Clopidogrel | Plavix |  |  |  |  |  |  |  |  |
| Disopyramide | Norpace |  |  |  |  |  |  |  |  |
| Dofetilide | Tikosyn |  |  |  |  |  |  |  |  |
| Dronedarone | Multaq |  |  |  |  |  |  |  |  |
| Eplerenone | Inspra |  |  |  |  |  |  |  |  |
| Flecainide | Tambocor |  |  |  |  |  |  |  |  |
| Ivabradine | Corlanor |  |  |  |  |  |  |  |  |
| Propafenone | Rythmol |  |  |  |  |  |  |  |  |
| Quinidine | Quinidex |  |  |  |  |  |  |  |  |
| **Neuropsychiatric** |  |  |  |  |  |  |  |  |  |
| Clozapine | Clozaril | FazaClo | Versacloz |  |  |  |  |  |  |
| Lurasidone | Latuda |  |  |  |  |  |  |  |  |
| Midazolam | Versed | Nayzilam | Seizalam |  |  |  |  |  |  |
| Pimozide | Orap |  |  |  |  |  |  |  |  |
| **Pulmonary Hypertension** |  |  |  |  |  |  |  |  |  |
| Sildenafil | Viagra | Revatio |  |  |  |  |  |  |  |
| Tadalafil | Cialis | Adcirca |  |  |  |  |  |  |  |
| Vardenafil | Levitra | Staxyn |  |  |  |  |  |  |  |
| **Miscellaneous** |  |  |  |  |  |  |  |  |  |
| Bosentan | Tracleer |  |  |  |  |  |  |  |  |
| Certain oncology-related medications^a^ |  |  |  |  |  |  |  |  |  |
| Ergotamine | Cafergot | Migergot |  |  |  |  |  |  |  |
| Dihydroergotamine | Migranal | D.H.E. 45 |  |  |  |  |  |  |  |
| Ergoloid Mesylates | Hydergine |  |  |  |  |  |  |  |  |
| Methylergonovine | Methergine |  |  |  |  |  |  |  |  |
| Lumacaftor/ivacaftor | Orkambi |  |  |  |  |  |  |  |  |
| St. John’s wort |  |  |  |  |  |  |  |  |  |
| Tolvaptan | Jynarque | Samsca |  |  |  |  |  |  |  |
| **Temporarily Withhold Concomitant Medication, if Clinically Appropriate** | | | | | | | | | |
| **Anticoagulants** |  |  |  |  |  |  |  |  |  |
| Rivaroxaban | Xarelto |  |  |  |  |  |  |  |  |
| **Anti-Infectives** |  |  |  |  |  |  |  |  |  |
| Erythromycin | Ery-Tab | Erythrocin | E.E.S. | Ilosone | EryPed |  |  |  |  |
| **BPH** |  |  |  |  |  |  |  |  |  |
| Alfuzosin | Uroxatral |  |  |  |  |  |  |  |  |
| Silodosin | Rapaflo |  |  |  |  |  |  |  |  |
| **Cardiovascular** |  |  |  |  |  |  |  |  |  |
| Aliskiren | Tekturna |  |  |  |  |  |  |  |  |
| Ranolazine | Ranexa |  |  |  |  |  |  |  |  |
| Ticagrelor | Brilinta |  |  |  |  |  |  |  |  |
| Vorapaxar | Zontivity |  |  |  |  |  |  |  |  |
| **Immunosuppressants** |  |  |  |  |  |  |  |  |  |
| Everolimus | Afinitor | Zortress |  |  |  |  |  |  |  |
| Sirolimus | Rapamune |  |  |  |  |  |  |  |  |
| Tacrolimus | Prograf | Astagraf XL | Envarsus XR |  |  |  |  |  |  |
| **Lipid-modifiers** |  |  |  |  |  |  |  |  |  |
| Atorvastatin | Lipitor |  |  |  |  |  |  |  |  |
| Lomitapide | Juxtapid |  |  |  |  |  |  |  |  |
| Lovastatin | Mevacor | Altoprev |  |  |  |  |  |  |  |
| Rosuvastatin | Crestor |  |  |  |  |  |  |  |  |
| Simvastatin | Zocor |  |  |  |  |  |  |  |  |
| **Migraine** |  |  |  |  |  |  |  |  |  |
| Eletriptan | Relpax |  |  |  |  |  |  |  |  |
| Rimegepant | Nurtec ODT |  |  |  |  |  |  |  |  |
| Ubrogepant | Ubrelvy |  |  |  |  |  |  |  |  |
| **Neuropsychiatric** |  |  |  |  |  |  |  |  |  |
| Daridorexant | Quviviq |  |  |  |  |  |  |  |  |
| Lemborexant | Dayvigo |  |  |  |  |  |  |  |  |
| Suvorexant | Belsomra |  |  |  |  |  |  |  |  |
| Triazolam | Halcion |  |  |  |  |  |  |  |  |
| **Erectile Dysfunction** |  |  |  |  |  |  |  |  |  |
| Avanafil | Stendra |  |  |  |  |  |  |  |  |
| **Respiratory** |  |  |  |  |  |  |  |  |  |
| Salmeterol | Serevent Diskus |  |  |  |  |  |  |  |  |
| **Miscellaneous** |  |  |  |  |  |  |  |  |  |
| Certain oncology-related medications^a^ |  |  |  |  |  |  |  |  |  |
| Colchicine | Colcrys | Mitigare | Gloperba |  |  |  |  |  |  |
| Finerenone | Kerendia |  |  |  |  |  |  |  |  |
| Flibanserin | Addyi |  |  |  |  |  |  |  |  |
| Naloxegol | Movantik |  |  |  |  |  |  |  |  |
| **Adjust Concomitant Medication Dose and Monitor for Adverse Effects** | | | | | | | | | |
| **Anticoagulants** |  |  |  |  |  |  |  |  |  |
| Apixaban | Eliquis |  |  |  |  |  |  |  |  |
| Dabigatran | Pradaxa |  |  |  |  |  |  |  |  |
| Edoxaban | Savaysa |  |  |  |  |  |  |  |  |
| **Anti-Infectives** |  |  |  |  |  |  |  |  |  |
| Clarithromycin | Biaxin |  |  |  |  |  |  |  |  |
| Itraconazole | Sporanox |  |  |  |  |  |  |  |  |
| Ketoconazole | Nizoral |  |  |  |  |  |  |  |  |
| Maraviroc | Selzentry |  |  |  |  |  |  |  |  |
| Rifabutin | Mycobutin |  |  |  |  |  |  |  |  |
| **BPH** |  |  |  |  |  |  |  |  |  |
| Tamsulosin | Flomax |  |  |  |  |  |  |  |  |
| **Cardiovascular** |  |  |  |  |  |  |  |  |  |
| Amlodipine | Norvasc |  |  |  |  |  |  |  |  |
| Cilostazol | Pletal |  |  |  |  |  |  |  |  |
| Digoxin | Lanoxin |  |  |  |  |  |  |  |  |
| Diltiazem | Cardizem | Cardizem CD | Cardizem LA | Cartia XT | Tiazac | Dilacor XR | Dilt-XR | Diltia XT | Matzim LA |
| Felodipine | Plendil |  |  |  |  |  |  |  |  |
| Nifedipine | Procardia | Adalat CC |  |  |  |  |  |  |  |
| Verapamil | Calan | Isoptin | Verelan | Covera-HS | |  |  |  |  |
| **Diabetes** |  |  |  |  |  |  |  |  |  |
| Saxagliptin | Onglyza |  |  |  |  |  |  |  |  |
| **Erectile Dysfunction** |  |  |  |  |  |  |  |  |  |
| Sildenafil | Viagra | Revatio |  |  |  |  |  |  |  |
| Tadalafil | Cialis | Adcirca |  |  |  |  |  |  |  |
| Vardenafil | Levitra | Staxyn |  |  |  |  |  |  |  |
| **Immunosuppressants** |  |  |  |  |  |  |  |  |  |
| Cyclosporine | Neoral | Sandimmune | Gengraf | Restasis | Cequa |  |  |  |  |
| Dexamethasone | Decadron | DexPak | Ozurdex | Hemady |  |  |  |  |  |
| Fedratinib | Inrebic |  |  |  |  |  |  |  |  |
| Ruxolitinib | Jakafi | Opzelura |  |  |  |  |  |  |  |
| Tofacitinib | Xeljanz |  |  |  |  |  |  |  |  |
| Upadacitinib | Rinvoq |  |  |  |  |  |  |  |  |
| **Migraine** |  |  |  |  |  |  |  |  |  |
| Almotriptan | Axert |  |  |  |  |  |  |  |  |
| **Neuropsychiatric** |  |  |  |  |  |  |  |  |  |
| Alprazolam | Xanax | Niravam |  |  |  |  |  |  |  |
| Aripiprazole | Abilify | Abilify Maintena | Aristada |  |  |  |  |  |  |
| Brexpiprazole | Rexulti |  |  |  |  |  |  |  |  |
| Buspirone | Buspar |  |  |  |  |  |  |  |  |
| Cariprazine | Vraylar |  |  |  |  |  |  |  |  |
| Chlordiazepoxide | Librium |  |  |  |  |  |  |  |  |
| Clobazam | Onfi | Sympazan |  |  |  |  |  |  |  |
| Clonazepam | Klonopin |  |  |  |  |  |  |  |  |
| Clorazepate | Tranxene |  |  |  |  |  |  |  |  |
| Diazepam | Valium | Diastat |  |  |  |  |  |  |  |
| Estazolam | Prosom |  |  |  |  |  |  |  |  |
| Flurazepam | Dalmane |  |  |  |  |  |  |  |  |
| Iloperidone | Fanapt |  |  |  |  |  |  |  |  |
| Lumateperone | Caplyta |  |  |  |  |  |  |  |  |
| Pimavanserin | Nuplazid |  |  |  |  |  |  |  |  |
| Quetiapine | Seroquel | Seroquel XR |  |  |  |  |  |  |  |
| Trazodone | Desyrel | Oleptro |  |  |  |  |  |  |  |
| **Pain** |  |  |  |  |  |  |  |  |  |
| Fentanyl | Duragesic | Actiq | Sublimaze | Lazanda | Abstral | Fentora |  |  |  |
| Hydrocodone | Hysingla ER | Zohydro ER |  |  |  |  |  |  |  |
| Oxycodone | OxyContin | Roxicodone | Xtampza ER | |  |  |  |  |  |
| **Pulmonary Hypertension** |  |  |  |  |  |  |  |  |  |
| Riociguat | Adempas |  |  |  |  |  |  |  |  |
| Miscellaneous |  |  |  |  |  |  |  |  |  |
| Certain oncology-related medications^a^ |  |  |  |  |  |  |  |  |  |
| Darifenacin | Enablex |  |  |  |  |  |  |  |  |
| Eluxadoline | Viberzi |  |  |  |  |  |  |  |  |
| Ivacaftor | Kalydeco |  |  |  |  |  |  |  |  |
| Solifenacin | Vesicare |  |  |  |  |  |  |  |  |
| Elexacaftor/tezacaftor/ivacaftor | Trikafta |  |  |  |  |  |  |  |  |
| Tezacaftor/ivacaftor | Symdeko |  |  |  |  |  |  |  |  |
| **Continue Concomitant Medication and Monitor for Adverse Effects** | | | | | | | | | |
| **Anticoagulants** |  |  |  |  |  |  |  |  |  |
| Warfarin | Coumadin | Jantoven |  |  |  |  |  |  |  |
| **Anti-Infectives** |  |  |  |  |  |  |  |  |  |
| Brincidofovir | Tembexa |  |  |  |  |  |  |  |  |
| Cobicistat | Tybost |  |  |  |  |  |  |  |  |
| Isavuconazole | Cresemba |  |  |  |  |  |  |  |  |
| Posaconazole | Noxafil |  |  |  |  |  |  |  |  |
| Voriconazole | Vfend |  |  |  |  |  |  |  |  |
| **BPH** |  |  |  |  |  |  |  |  |  |
| Doxazosin | Cardura |  |  |  |  |  |  |  |  |
| Terazosin | Hytrin |  |  |  |  |  |  |  |  |
| **Diabetes** |  |  |  | |  |  |  |  |  |
| Glyburide | Diabeta | Glynase | Micronase | |  |  |  |  |  |
| **Cardiovascular** |  |  |  |  |  |  |  |  |  |
| Mexiletine | Mexitil |  |  |  |  |  |  |  |  |
| Sacubitril | Entresto |  |  |  |  |  |  |  |  |
| Valsartan | Diovan | Prexxartan |  |  |  |  |  |  |  |
| **Migraine** |  |  |  |  |  |  |  |  |  |
| Zolmitriptan | Zomig |  |  |  |  |  |  |  |  |
| **Neuropsychiatric** |  |  |  |  |  |  |  |  |  |
| Haloperidol | Haldol |  |  |  |  |  |  |  |  |
| Hydroxyzine | Atarax | Vistaril |  |  |  |  |  |  |  |
| Mirtazapine | Remeron |  |  |  |  |  |  |  |  |
| Risperidone | Risperdal |  |  |  |  |  |  |  |  |
| Ziprasidone | Geodon |  |  |  |  |  |  |  |  |
| Zolpidem | Ambien | Ambien CR | Edluar | Intermezzo | Zolpimist |  |  |  |  |
| **Pain** |  |  |  |  |  |  |  |  |  |
| Buprenorphine | Subutex | Suboxone | Sublocade | Belbuca | Butrans |  |  |  |  |
| Hydromorphone | Dilaudid | Exalgo |  |  |  |  |  |  |  |
| Methadone | Dolophine | Methadose |  |  |  |  |  |  |  |
| Morphine | MS Contin | Duramorph | Kadian | Arymo ER | Morphabond ER | Roxanol |  |  |  |
| Tramadol | Ultram | ConZip | Qdolo |  |  |  |  |  |  |
| **Miscellaneous** |  |  |  |  |  |  |  |  |  |
| Oxybutynin | Ditropan | Ditropan XL | Oxytrol | Gelnique |  |  |  |  |  |
| Certain oncology-related medications^a^ |  |  |  |  |  |  |  |  |  |
| Certain conjugated monoclonal antibodies^b^ |  |  |  |  |  |  |  |  |  |
| **Certain oncology-related medications** | | | | | | | | | |
| Apalutamide | Erleada |  |  |  |  |  |  |  |  |
| Abemaciclib | Verzenio |  |  |  |  |  |  |  |  |
| Ceritinib | Zykadia |  |  |  |  |  |  |  |  |
| Dasatinib | Sprycel |  |  |  |  |  |  |  |  |
| Encorafenib | Braftovi |  |  |  |  |  |  |  |  |
| Ibrutinib | Imbruvica |  |  |  |  |  |  |  |  |
| Ivosidenib | Tibsovo |  |  |  |  |  |  |  |  |
| Neratinib | Nerlynx |  |  |  |  |  |  |  |  |
| Nilotinib | Tasigna |  |  |  |  |  |  |  |  |
| Venetoclax | Venclexta |  |  |  |  |  |  |  |  |
| Vinblastine | Velban |  |  |  |  |  |  |  |  |
| Vincristine | Oncovin |  |  |  |  |  |  |  |  |
| Abemaciclib | Verzenio |  |  |  |  |  |  |  |  |
| Acalabrutinib | Calquence |  |  |  |  |  |  |  |  |
| Apalutamide | Erleada |  |  |  |  |  |  |  |  |
| Avapritinib | Ayvakit |  |  |  |  |  |  |  |  |
| Axitinib | Inlyta |  |  |  |  |  |  |  |  |
| Bexarotene | Targretin |  |  |  |  |  |  |  |  |
| Bortezomib | Velcade |  |  |  |  |  |  |  |  |
| Bosutinib | Bosulif |  |  |  |  |  |  |  |  |
| Brigatinib | Alunbrig |  |  |  |  |  |  |  |  |
| Cabozantinib | Cabometyx | Cometriq |  |  |  |  |  |  |  |
| Ceritinib | Zykadia |  |  |  |  |  |  |  |  |
| Cobimetinib | Cotellic |  |  |  |  |  |  |  |  |
| Crizotinib | Xalkori |  |  |  |  |  |  |  |  |
| Dabrafenib | Tafinlar |  |  |  |  |  |  |  |  |
| Darolutamide | Nubeqa |  |  |  |  |  |  |  |  |
| Dasatinib | Sprycel |  |  |  |  |  |  |  |  |
| Dexamethasone | Decadron | DexPak | Baycadron | Maxidex | Ozurdex | Hexadrol | Zodex |  |  |
| Duvelisib | Copiktra |  |  |  |  |  |  |  |  |
| Enasidenib | Idhifa |  |  |  |  |  |  |  |  |
| Encorafenib | Braftovi |  |  |  |  |  |  |  |  |
| Entrectinib | Rozlytrek |  |  |  |  |  |  |  |  |
| Enzalutamide | Xtandi |  |  |  |  |  |  |  |  |
| Erdafitinib | Balversa |  |  |  |  |  |  |  |  |
| Erlotinib | Tarceva |  |  |  |  |  |  |  |  |
| Etoposide | Toposar | VePesid |  |  |  |  |  |  |  |
| Everolimus | Afinitor |  |  |  |  |  |  |  |  |
| Fedratinib | Inrebic |  |  |  |  |  |  |  |  |
| Flutamide | Eulexin |  |  |  |  |  |  |  |  |
| Fostamatinib | Tavalisse |  |  |  |  |  |  |  |  |
| Gefitinib | Iressa |  |  |  |  |  |  |  |  |
| Gilteritinib | Xospata |  |  |  |  |  |  |  |  |
| Glasdegib | Daurismo |  |  |  |  |  |  |  |  |
| Ibrutinib | Imbruvica |  |  |  |  |  |  |  |  |
| Idelalisib | Zydelig |  |  |  |  |  |  |  |  |
| Imatinib | Gleevec |  |  |  |  |  |  |  |  |
| Infigratinib | Truseltiq |  |  |  |  |  |  |  |  |
| Isotretinoin | Absorica | Claravis | Amnesteem | Zenatane | Myorisan |  |  |  |  |
| Ivosidenib | Tibsovo |  |  |  |  |  |  |  |  |
| Lapatinib | Tykerb |  |  |  |  |  |  |  |  |
| Larotrectinib | Vitrakvi |  |  |  |  |  |  |  |  |
| Lorlatinib | Lorbrena |  |  |  |  |  |  |  |  |
| Midostaurin | Rydapt |  |  |  |  |  |  |  |  |
| Mitotane | Lysodren |  |  |  |  |  |  |  |  |
| Mobocertinib | Exkivity |  |  |  |  |  |  |  |  |
| Neratinib | Nerlynx |  |  |  |  |  |  |  |  |
| Nilotinib | Tasigna |  |  |  |  |  |  |  |  |
| Olaparib | Lynparza |  |  |  |  |  |  |  |  |
| Palbociclib | Ibrance |  |  |  |  |  |  |  |  |
| Panobinostat | Farydak |  |  |  |  |  |  |  |  |
| Pazopanib | Votrient |  |  |  |  |  |  |  |  |
| Pemigatinib | Pemazyre |  |  |  |  |  |  |  |  |
| Pexidartinib | Turalio |  |  |  |  |  |  |  |  |
| Ponatinib | Iclusig |  |  |  |  |  |  |  |  |
| Pralsetinib | Gavreto |  |  |  |  |  |  |  |  |
| Regorafenib | Stivarga |  |  |  |  |  |  |  |  |
| Relugolix | Orgovyx |  |  |  |  |  |  |  |  |
| Ribociclib | Kisqali |  |  |  |  |  |  |  |  |
| Ripretinib | Qinlock |  |  |  |  |  |  |  |  |
| Rucaparib | Rubraca |  |  |  |  |  |  |  |  |
| Ruxolitinib | Jakafi |  |  |  |  |  |  |  |  |
| Selinexor | Xpovio |  |  |  |  |  |  |  |  |
| Selpercatinib | Retevmo |  |  |  |  |  |  |  |  |
| Sonidegib | Odomzo |  |  |  |  |  |  |  |  |
| Sorafenib | Nexavar |  |  |  |  |  |  |  |  |
| Sotorasib | Lumakras |  |  |  |  |  |  |  |  |
| Sunitinib | Sutent |  |  |  |  |  |  |  |  |
| Talazoparib | Talzenna |  |  |  |  |  |  |  |  |
| Tepotinib | Tepmetko |  |  |  |  |  |  |  |  |
| Umbralisib | Ukoniq |  |  |  |  |  |  |  |  |
| Vandetanib | Caprelsa |  |  |  |  |  |  |  |  |
| Vemurafenib | Zelboraf |  |  |  |  |  |  |  |  |
| Venetoclax | Venclexta |  |  |  |  |  |  |  |  |
| Vinblastine | Velban |  |  |  |  |  |  |  |  |
| Vincristine | Oncovin |  |  |  |  |  |  |  |  |
| Zanubrutinib | Brukinsa |  |  |  |  |  |  |  |  |
| **Conjugated monoclonal antibodies** |  |  |  |  |  |  |  |  |  |
| Ado-trastuzumab emtansine | Kadcyla |  |  |  |  |  |  |  |  |
| Mirvetuximab soravtansine | Elahere |  |  |  |  |  |  |  |  |
| Brentuximab vedotin | Adcetris |  |  |  |  |  |  |  |  |
| Enfortumab vedotin | Padcev |  |  |  |  |  |  |  |  |
| Polatuzumab vedotin | Polivy |  |  |  |  |  |  |  |  |
| Tisotumab vedotin | Tivdak |  |  |  |  |  |  |  |  |

Abbreviations: BPH = Benign Prostatic Hyperplasia, CC= Continuous Control, CD = Controlled Delivery, CR = Controlled Release, D.H.E 45= **Dihydroergotamine mesylate injection (45 = 1 mg/mL strength), E.E.S=** erythromycin ethylsuccinate **(45 = 1 mg/mL strength),** ER = Extended Release, LA=Long Acting, ODT = Orally Disintegrating Tablet, XL = Extended Release (manufacturer-specific designation), XR = Extended Release, XT = Extended Release,

CD = Controlled Delivery, CC = Continuous Control.

^a,b^ Certain oncology-related medications and monoclonal antibodies referenced under the Miscellaneous category are listed at the end of the table.

**Supplementary Figure S2. Frequency and percentage of nirmatrelvir/ritonavir administrations by month-year, 2022-2025.**

Note: The numbers at the top of the bars represent the monthly percentage of total nirmatrelvir/ritonavir administrations during the study period.

**Supplementary Table S2. Characteristics of long-term care facility residents administered nirmatrelvir/ritonavir by the occurrence of a potential drug-drug interaction.**

| **Characteristics** | **Overall**  N=48,260 | **No potential DDI with nirmatrelvir/ritonavir**  N=6,803 | **≥1 potential DDI with nirmatrelvir/ritonavir**  N=41,457 |
| --- | --- | --- | --- |
| Age in years, median (Q1, Q3) ^a^ | 80.0 (72.0, 87.0) | 82.0 (74.0, 89.0) | 79.0 (71.0, 87.0) |
| Age category, years, N (%) |  |  |  |
| ≤ 70 | 9,719 (20.1) | 1,060 (15.6) | 8,659 (20.9) |
| 71-75 | 6,616 (13.7) | 778 (11.4) | 5,838 (14.1) |
| 76-80 | 7,544 (15.6) | 957 (14.1) | 6,587 (15.9) |
| 81-85 | 7,833 (16.2) | 1,172 (17.2) | 6,661 (16.1) |
| ≥ 86 | 13,419 (27.8) | 2,484 (36.5) | 10,935 (26.4) |
| Missing | 3,129 (6.5) | 352 (5.2) | 2,777 (6.7) |
| Sex, N (%) |  |  |  |
| Female | 27,755 (57.5) | 4,297 (63.2) | 23,458 (56.6) |
| Male | 17,376 (36) | 2,154 (31.7) | 15,222 (36.7) |
| Missing | 3,129 (6.5) | 352 (5.2) | 2,777 (6.7) |
| Race, N (%) |  |  |  |
| American Indian/ Alaska Native | 177 (0.4) | 33 (0.5) | 144 (0.3) |
| Asian/Pacific Islander | 849 (1.8) | 153 (2.2) | 696 (1.7) |
| Black (or African-American) | 4,414 (9.1) | 485 (7.1) | 3,929 (9.5) |
| Hispanic | 2,282 (4.7) | 327 (4.8) | 1,955 (4.7) |
| Missing | 3,129 (6.5) | 352 (5.2) | 2,777 (6.7) |
| Non-Hispanic White | 36,791 (76.2) | 5,363 (78.8) | 31,428 (75.8) |
| Other | 261 (0.5) | 38 (0.6) | 223 (0.5) |
| Unknown | 357 (0.7) | 52 (0.8) | 305 (0.7) |
| Comorbid conditions, N (%) ^b^ |  |  |  |
| Diagnosed Dementia | 10,085 (20.9) | 1,425 (21.0) | 8,660 (21.0) |
| Diabetes | 15,157 (31.4) | 2,154 (31.7) | 13,003 (31.4) |
| Chronic Heart Failure | 11,035 (22.9) | 1,557 (22.9) | 9,478 (22.9) |
| Chronic Kidney Disease | 14,450 (30.0) | 2,068 (30.4) | 12,382 (29.9) |
| Stays in LTCF ^c^ |  |  |  |
| Time spent in LTCF, days, median (Q1, Q3) | 13 (0, 47) | 12 (0, 44) | 13 (0, 47) |
| Long stays (>100 days in LTCF), N (%) | 7,797 (16.2) | 1,068 (15.7) | 6,729 (16.2) |
| Polypharmacy ^d^ |  |  |  |
| Total unique medications administered in the LTCF before nirmatrelvir/ritonavir administration, mean (SD) | 1.8 (5.8) | 1.7 (5.6) | 1.8 (5.8) |

Abbreviations: DDI=Drug-Drug Interaction, LTCF=Long-Term Care Facility, N=Number, %=Percentage, Q1=Lower Quartile, Q3=Upper Quartile, SD=Standard Deviation.

^a^ Descriptive statistic for age at the time of first nirmatrelvir/ritonavir administration during the study period is reported among LTCF residents with non-missing values.

^b^ Comorbid conditions were identified using diagnosis codes recorded in the Conditions domain of the Long-Term Care Data Cooperative and were defined based on the presence of at least one corresponding diagnosis.

^c^ Time in long-term care was defined as the number of days of residence prior to the first nirmatrelvir/ritonavir administration during the study period.

^d^ Total medications reflect the number of distinct medications administered in the LTCF during the 180 days prior to each resident’s first nirmatrelvir/ritonavir administration during the study period.

**Supplementary Table S3. Additional clinical characteristics and drug-drug interaction burden.**

| **Characteristic** | **N (%)** |
| --- | --- |
| Potential DDIs |  |
| ≥2 potential DDIs | 29,397 (60.9) |
| ≥3 potential DDIs | 17,042 (35.3) |
| Number of potential DDIs, mean (SD) | 2.1 (1.5) |
| CYP3A4 Inhibitors (excluding nirmatrelvir/ritonavir) |  |
| Any additional CYP3A4 inhibitor | 4,011 (8.3) |
| Amiodarone | 1,147 (2.4) |
| Erythromycin | 165 (0.3) |
| Diltiazem | 1,855 (3.8) |
| Ketoconazole | 757 (1.6) |
| Verapamil | 241 (0.5) |

Abbreviations: DDIs=Drug-Drug Interaction, CYP3A4=Cytochrome P450 3A4, N=Number, %=Percentage, SD=Standard Deviation.

A potential DDI was defined as the same-day co-administration of nirmatrelvir/ritonavir with a potentially interacting medication.

CYP3A4 inhibitors were identified based on same-day co-administration with nirmatrelvir/ritonavir and therefore represent a subset of potential DDIs.

Percentages are calculated using the full cohort of long-term care facility residents treated with nirmatrelvir/ritonavir (N=48,260).

**Supplementary Table S4: Prevalence and median duration of exposure to 142 potentially interacting medications.**

| **Drug** | **Prevalence (95% CLs) %** | **Median days of exposure (Q1, Q3)** |
| --- | --- | --- |
| Abemaciclib | N/A | N/A |
| Acalabrutinib | N/A | N/A |
| Alfuzosin | 0.16 (0.12, 0.19) | 5.0 (2.0, 6.0) |
| Aliskiren | N/A | N/A |
| Alprazolam | 2.67 (2.53, 2.82) | 5.0 (3.0, 6.0) |
| Amiodarone | 2.38 (2.24, 2.52) | 5.0 (2.0, 6.0) |
| Amlodipine | 21.56 (21.19, 21.93) | 5.0 (5.0, 6.0) |
| Apalutamide | N/A | N/A |
| Apixaban | 15.93 (15.60, 16.26) | 5.0 (4.0, 6.0) |
| Aripiprazole | 1.90 (1.78, 2.03) | 5.0 (5.0, 6.0) |
| Atorvastatin | 28.06 (27.66, 28.47) | 5.0 (2.0, 6.0) |
| Axitinib | N/A | N/A |
| Bosentan | N/A | N/A |
| Bosutinib | N/A | N/A |
| Brexpiprazole | 0.16 (0.12, 0.20) | 5.0 (5.0, 6.0) |
| Bromocriptin | 0.06 (0.04, 0.09) | 5.5 (5.0, 8.0) |
| Buprenorphine | 0.36 (0.31, 0.42) | 3.5 (1.0, 5.0) |
| Buspirone | 6.16 (5.95, 6.38) | 5.0 (5.0, 6.0) |
| Cabozantinib | N/A | N/A |
| Carbamazepine | 0.50 (0.44, 0.57) | 5.0 (2.0, 6.0) |
| Cariprazine | 0.12 (0.09, 0.16) | 5.0 (5.0, 6.0) |
| Chlordiazepoxide | 0.04 (0.02, 0.06) | 5.0 (5.0, 6.0) |
| Cilostazol | 0.35 (0.30, 0.40) | 5.0 (5.0, 6.0) |
| Clarithromycin | 0.08 (0.06, 0.11) | 5.0 (4.5, 5.0) |
| Clobazam | 0.06 (0.04, 0.09) | 5.0 (3.0, 6.0) |
| Clonazepam | 2.48 (2.35, 2.63) | 5.0 (4.0, 6.0) |
| Clopidogrel | 8.78 (8.53, 9.04) | 5.0 (5.0, 6.0) |
| Clorazepate | 0.02 (0.01, 0.04) | 5.0 (2.5, 5.5) |
| Clozapine | 0.28 (0.24, 0.34) | 5.0 (4.0, 6.0) |
| Cobicistat | N/A | N/A |
| Colchicine | 0.40 (0.34, 0.46) | 4.0 (1.0, 5.0) |
| Cyclosporine | 0.86 (0.78, 0.94) | 5.0 (5.0, 6.0) |
| Dabigatran | 0.40 (0.35, 0.46) | 5.0 (3.0, 6.0) |
| Dabrafenib | N/A | N/A |
| Daridorexant | N/A | N/A |
| Darifenacin | N/A | N/A |
| Darolutamide | N/A | N/A |
| Dasatinib | N/A | N/A |
| Dexamethasone | 6.73 (6.50, 6.95) | 5.0 (3.0, 5.0) |
| Diazepam | 0.63 (0.56, 0.71) | 5.0 (3.0, 6.0) |
| Digoxin | 1.40 (1.30, 1.51) | 5.0 (3.0, 6.0) |
| Diltiazem | 3.84 (3.67, 4.02) | 5.0 (5.0, 6.0) |
| Disopyramide | N/A | N/A |
| Dofetilide | 0.06 (0.04, 0.09) | 5.0 (3.0, 6.0) |
| Doxazosin | 0.99 (0.90, 1.08) | 5.0 (5.0, 6.0) |
| Dronedarone | 0.08 (0.06, 0.11) | 5.0 (3.0, 6.0) |
| Edoxaban | N/A | N/A |
| Eletriptan | N/A | N/A |
| Eluxadoline | N/A | N/A |
| Enzalutamide | 0.03 (0.02, 0.05) | 6.0 (1.0, 7.0) |
| Eplerenone | 0.06 (0.04, 0.08) | 4.5 (1.5, 6.0) |
| Erdafitinib | N/A | N/A |
| Erythromycin | 0.34 (0.29, 0.40) | 5.0 (3.0, 6.0) |
| Everolimus | N/A | N/A |
| Felodipine | 0.09 (0.07, 0.12) | 5.0 (4.0, 6.0) |
| Fentanyl | 0.71 (0.63, 0.79) | 2.0 (1.0, 2.0) |
| Finerenone | N/A | N/A |
| Flecainide | 0.19 (0.16, 0.24) | 5.0 (2.0, 6.0) |
| Fostamatinib | N/A | N/A |
| Glecaprevir/ pibrentasvir | N/A | N/A |
| Glyburide | 0.11 (0.08, 0.14) | 5.0 (4.0, 6.0) |
| Haloperidol | 0.67 (0.60, 0.75) | 5.0 (3.0, 6.0) |
| Hydrocodone | 9.46 (9.20, 9.72) | 4.0 (2.0, 5.0) |
| Hydromorphone | 0.49 (0.43, 0.56) | 5.0 (2.0, 6.0) |
| Hydroxyzine | 2.17 (2.04, 2.31) | 5.0 (2.0, 6.0) |
| Ibrutinib | N/A | N/A |
| Iloperidone | N/A | N/A |
| Imatinib | 0.03 (0.02, 0.05) | 5.0 (5.0, 6.0) |
| Isavuconazole | N/A | N/A |
| Itraconazole | N/A | N/A |
| Ivabradine | 0.03 (0.02, 0.05) | 4.0 (1.5, 5.5) |
| Ketoconazole | 1.57 (1.46, 1.68) | 2.0 (1.0, 5.0) |
| Lorlatinib | N/A | N/A |
| Lovastatin | 0.48 (0.42, 0.54) | 3.0 (1.0, 5.0) |
| Lumateperone | N/A | N/A |
| Lurasidone | 0.16 (0.12, 0.19) | 5.0 (1.0, 6.0) |
| Maraviroc | N/A | N/A |
| Methadone | 0.45 (0.39, 0.52) | 5.0 (5.0, 6.0) |
| Mexiletine | 0.04 (0.03, 0.06) | 5.0 (2.0, 6.0) |
| Midazolam | N/A | N/A |
| Mirtazapine | 10.12 (9.85, 10.39) | 5.0 (5.0, 6.0) |
| Morphine | 1.92 (1.80, 2.05) | 4.0 (2.0, 5.0) |
| Naloxegol | 0.18 (0.14, 0.22) | 5.0 (2.0, 6.0) |
| Nifedipine | 1.53 (1.42, 1.64) | 5.0 (4.0, 6.0) |
| Nilotinib | N/A | N/A |
| Olaparib | N/A | N/A |
| Oxybutynin | 3.50 (3.34, 3.67) | 5.0 (5.0, 6.0) |
| Oxycodone | 8.56 (8.31, 8.81) | 4.0 (2.0, 5.0) |
| Palbociclib | N/A | N/A |
| Pemigatinib | N/A | N/A |
| Phenobarbital | 0.17 (0.13, 0.21) | 5.0 (2.0, 6.0) |
| Phenytoin | 0.52 (0.45, 0.58) | 5.0 (2.0, 6.0) |
| Pimavanserin | 0.14 (0.11, 0.17) | 5.0 (3.0, 6.0) |
| Pimozide | N/A | N/A |
| Posaconazole | N/A | N/A |
| Primidone | 0.79 (0.71, 0.87) | 5.0 (2.0, 6.0) |
| Propafenone | 0.06 (0.04, 0.08) | 5.0 (2.0, 6.0) |
| Quetiapine | 5.60 (5.40, 5.81) | 5.0 (4.0, 6.0) |
| Quinidine | 0.28 (0.24, 0.33) | 5.0 (3.0, 6.0) |
| Ranolazine | 0.47 (0.42, 0.54) | 5.0 (2.0, 6.0) |
| Relugolix | N/A | N/A |
| Rifabutin | N/A | N/A |
| Rifampin | 0.03 (0.02, 0.05) | 4.5 (1.5, 5.5) |
| Rimegepant | 0.03 (0.02, 0.05) | 1.0 (1.0, 2.0) |
| Riociguat | N/A | N/A |
| Risperidone | 3.17 (3.02, 3.33) | 5.0 (5.0, 6.0) |
| Rivaroxaban | 2.50 (2.36, 2.64) | 5.0 (1.0, 5.0) |
| Rosuvastatin | 3.06 (2.91, 3.22) | 5.0 (2.0, 6.0) |
| Ruxolitinib | N/A | N/A |
| Sacubitril | 1.41 (1.30, 1.52) | 5.0 (5.0, 6.0) |
| Salmeterol | 3.37 (3.21, 3.53) | 5.0 (4.0, 6.0) |
| Saxagliptin | 0.04 (0.03, 0.07) | 5.0 (4.0, 6.0) |
| Selinexor | N/A | N/A |
| Sildenafil | 0.06 (0.04, 0.08) | 5.0 (2.0, 6.0) |
| Silodosin | 0.08 (0.06, 0.11) | 5.0 (2.5, 6.0) |
| Simvastatin | 2.82 (2.68, 2.97) | 4.0 (1.0, 5.0) |
| Sirolimus | N/A | N/A |
| Solifenacin | 0.50 (0.44, 0.56) | 5.0 (5.0, 6.0) |
| Sonidegib | N/A | N/A |
| Sotorasib | N/A | N/A |
| Suvorexant | 0.05 (0.03, 0.07) | 5.0 (3.0, 5.5) |
| Tacrolimus | 0.18 (0.14, 0.22) | 5.0 (3.0, 6.0) |
| Tadalafil | 0.05 (0.03, 0.07) | 5.0 (2.5, 6.0) |
| Tamsulosin | 11.81 (11.52, 12.10) | 5.0 (2.0, 6.0) |
| Terazosin | 0.68 (0.61, 0.76) | 5.0 (5.0, 6.0) |
| Ticagrelor | 0.27 (0.23, 0.32) | 5.0 (1.0, 5.0) |
| Tofacitinib | N/A | N/A |
| Tolvaptan | N/A | N/A |
| Tramadol | 7.41 (7.18, 7.65) | 5.0 (2.0, 6.0) |
| Trazodone | 10.81 (10.53, 11.09) | 5.0 (5.0, 6.0) |
| Triazolam | N/A | N/A |
| Ubrogepant | N/A | N/A |
| Upadacitinib | N/A | N/A |
| Valsartan | 1.30 (1.20, 1.40) | 5.0 (5.0, 6.0) |
| Venetoclax | N/A | N/A |
| Verapamil | 0.52 (0.46, 0.59) | 5.0 (5.0, 6.0) |
| Voriconazole | N/A | N/A |
| Warfarin | 1.79 (1.67, 1.91) | 5.0 (3.0, 5.0) |
| Zanubrutinib | N/A | N/A |
| Ziprasidone | 0.32 (0.27, 0.37) | 5.0 (5.0, 6.0) |
| Zolmitriptan | N/A | N/A |
| Zolpidem | 0.83 (0.75, 0.91) | 5.0 (3.0, 6.0) |

Abbreviations: CLs=Confidence Limits, Q1=Lower Quartile, Q3=Upper Quartile.

Note: Cells showing N/A have been suppressed to comply with the Centers for Medicare and Medicaid Services Small Cell Suppression Policy.
